# Pulmonary haemodynamics by echocardiography over 3 days of acclimatization in lowlanders with chronic obstructive pulmonary disease travelling to 3100 m of high altitude

**DOI:** 10.1093/ehjopen/oeaf017

**Published:** 2025-03-04

**Authors:** Konstantinos Bitos, Julian Müller, Adilet Omuralieva, Simon R Schneider, Mona Lichtblau, Stéphanie Saxer, Felix C Tanner, Michael Furian, Maamed Mademilov, Talant Sooronbaev, Konrad E Bloch, Silvia Ulrich

**Affiliations:** Department of Cardiology, University Hospital of Zurich, Raemistrasse 100, Zurich 8091, Switzerland; Department of Pulmonology, University Hospital of Zurich, Raemistrasse 100, Zurich 8091, Switzerland; Faculty of Medicine, University of Zurich, Pestalozzistrasse 3, Zurich 8032, Switzerland; Department of Cardiology, National Center of Cardiology and Internal Medicine Named after Academician M. Mirrakhimov, Bishkek, Kyrgyzstan; Department of Pulmonology, University Hospital of Zurich, Raemistrasse 100, Zurich 8091, Switzerland; Department of Pulmonology, University Hospital of Zurich, Raemistrasse 100, Zurich 8091, Switzerland; Faculty of Medicine, University of Zurich, Pestalozzistrasse 3, Zurich 8032, Switzerland; Department of Pulmonology, University Hospital of Zurich, Raemistrasse 100, Zurich 8091, Switzerland; Department of Health, Eastern Switzerland University of Applied Sciences, Rosenbergstrasse 59, St. Gallen 9000, Switzerland; Department of Cardiology, University Hospital of Zurich, Raemistrasse 100, Zurich 8091, Switzerland; Department of Pulmonology, University Hospital of Zurich, Raemistrasse 100, Zurich 8091, Switzerland; Faculty of Medicine, University of Zurich, Pestalozzistrasse 3, Zurich 8032, Switzerland; Swiss-Kyrgyz High Altitude Medicine and Research Initiative, Zurich, Switzerland, and Bishkek, Kyrgyzstan; Department of Cardiology, National Center of Cardiology and Internal Medicine Named after Academician M. Mirrakhimov, Bishkek, Kyrgyzstan; Swiss-Kyrgyz High Altitude Medicine and Research Initiative, Zurich, Switzerland, and Bishkek, Kyrgyzstan; Department of Cardiology, National Center of Cardiology and Internal Medicine Named after Academician M. Mirrakhimov, Bishkek, Kyrgyzstan; Swiss-Kyrgyz High Altitude Medicine and Research Initiative, Zurich, Switzerland, and Bishkek, Kyrgyzstan; Department of Pulmonology, University Hospital of Zurich, Raemistrasse 100, Zurich 8091, Switzerland; Faculty of Medicine, University of Zurich, Pestalozzistrasse 3, Zurich 8032, Switzerland; Swiss-Kyrgyz High Altitude Medicine and Research Initiative, Zurich, Switzerland, and Bishkek, Kyrgyzstan; Department of Pulmonology, University Hospital of Zurich, Raemistrasse 100, Zurich 8091, Switzerland; Faculty of Medicine, University of Zurich, Pestalozzistrasse 3, Zurich 8032, Switzerland; Swiss-Kyrgyz High Altitude Medicine and Research Initiative, Zurich, Switzerland, and Bishkek, Kyrgyzstan

**Keywords:** Pulmonary haemodynamics, Acclimatization, High altitude

## Abstract

**Aims:**

Patients with chronic obstructive pulmonary disease experience an increase in systolic pulmonary artery pressure (sPAP) when exposed to high altitude with an unclear acclimatization. We investigated the effects of acute ascent to 3100 m on pulmonary haemodynamics of patients with chronic obstructive pulmonary disease and their acclimatization during a 3-day stay at high altitude.

**Methods and results:**

In this prospective, interventional study, stable, normocapnic patients with chronic obstructive pulmonary disease, with FEV_1_ 40–80%predicted and SpO_2_ ≥ 92%, residing at low altitude and staying for 3 days/nights at 3100 m without adverse events, were included. Echocardiography was performed at 760 m, directly after arrival at 3100 m (HA1) and the two following days (HA2/HA3). The primary outcome was the change in sPAP at different time points. Additionally, cardiac output (CO), tricuspid annular plane systolic excursion (TAPSE), and other echocardiographic parameters were measured. Thirty-eight patients with chronic obstructive pulmonary disease (37% females), aged (mean ± SD) 55 ± 10years, with FEV_1_ 63 ± 12%predicted, were included. After acute ascent to 3100 m vs. 760 m, sPAP increased by 12 mmHg [95% confidence interval (CI): 9–15, *P* < 0.001], total pulmonary resistance (sPAP/CO) increased by 2 WU (1–3, *P* = 0.001), and TAPSE/sPAP decreased by −0.6 mm/mmHg (−0.9 to −0.2, *P* = 0.002). Right atrial pressure and CO were unchanged. At HA3 compared to HA1, sPAP decreased by −4 mmHg (−7 to −1, *P* = 0.008); no significant changes in further echocardiographic parameters were observed.

**Conclusion:**

In stable patients with chronic obstructive pulmonary disease travelling to and staying at 3100 m for 3 days/nights without adverse events, sPAP initially increased, along with an increased pulmonary resistance and a reduced right ventricular-arterial coupling reflected by a lower TAPSE/sPAP. Whereas sPAP steadily decreased during acclimatization, other echocardiographic parameters remained unchanged.

## Introduction

Chronic obstructive pulmonary disease has a high prevalence in the general population and represents one of the top three aetiologies of mortality worldwide, especially in low- and middle-income countries.^1,2^ The clinical presentation is characterized by chronic respiratory symptoms, e.g. dyspnoea, productive cough, and exercise intolerance, caused by chronic airflow obstruction and airway inflammation.^3^ A common complication in patients with chronic obstructive pulmonary disease is the development of pulmonary hypertension (PH) due to an increased ventilation-perfusion mismatch and intrapulmonary shunt, a reduced mixed-venous partial pressure of oxygen,^4^ hypoxic pulmonary vasoconstriction, and a decrease of the pulmonary capillary bed. Once PH is established in patients with chronic obstructive pulmonary disease, it progresses with around 1 mmHg per year.^5^

Taking into consideration these alternations of pulmonary haemodynamics, patients with chronic obstructive pulmonary disease, who travel to high altitude (HA), may have a higher risk to develop exaggerated PH when being exposed to hypobaric hypoxia. Studies have reported that patients with chronic obstructive pulmonary disease tolerate the acute exposure to moderate HA, but they suffer from increased rate of acute mountain sickness and reduced exercise capacity and may need supplemental oxygen therapy due to severe hypoxaemia.^6–10^ Furthermore, in patients with chronic obstructive pulmonary disease residing at HA, PH has been shown to be associated with an altered exercise capacity and gas exchange disturbances.^11^ The tricuspid regurgitation pressure gradient (TRPG), measured by echocardiography, can be used to estimate the systolic pulmonary artery pressure (sPAP). Data from randomized controlled clinical trials found exaggerated increase of sPAP in patients with chronic obstructive pulmonary disease travelling to HA.^12–15^ However, the impact of acclimatization during a 3-day stay at HA on pulmonary haemodynamics and right heart function in patients with chronic obstructive pulmonary disease remains unclear.

Therefore, the aim of the current study was to investigate the acclimatization effect on pulmonary haemodynamics and right heart function in patients with chronic obstructive pulmonary disease who travel to HA and stay there for 3 days without experiencing any other altitude related adverse health event (ARAHE), to provide a better insight to the underlying pathophysiological background.

## Methods

### Study design

The current study was performed as part of an umbrella study which was a randomized, placebo-controlled, double-blinded trial evaluating the effect of acetazolamide on right heart function in lowlanders with chronic obstructive pulmonary disease travelling to HA (3100 m) and developing early symptoms and/or signs of impending altitude illness (Clinicaltrials.gov: NCT04913389). Patients who have been recruited for the umbrella study and completed a 3-day stay at 3100 m, but did not experience any ARAHE, were excluded from the main study and, therefore, were selected for the present analysis. The study took place in the Kyrgyz Republic as project of the Swiss-Kyrgyz High Altitude Medicine and Research Initiative from May to August 2021. The baseline measurements were performed in Bishkek at 760 m [low altitude (LA)]. The patients were then transferred by bus to Tuja Ashu HA clinic, at 3100 m, where they stayed for three days and two nights. The protocol of the main study has been approved by the ethics committee of the National Center of Cardiology and Internal Medicine (NCCIM) in Bishkek, Kyrgyz Republic (Nr. 01-7/181), and the study has been registered at ClinicalTrials.gov (NCT04915365).

### Consent

The study complies with the Declaration of Helsinki, and all patients who participated provided written informed consent.

### Patients

In the current study, we included male and female from 35 to 75 years of age, who were born, raised, and currently living at LA (<800 m), with moderate to severe chronic obstructive pulmonary disease defined as FEV_1_ 40–80%predicted.^16^ For inclusion of this analysis, patients need to have a sufficient echo quality and had to complete the 3-day stay at 3100 m, without experiencing early signs of ARAHE (SpO_2_ < 85%, or moderate symptoms of acute mountain sickness) that qualified the patients to be included into the main trial mentioned above. Any ARAHE defined as any of the following, acute mountain sickness (Lake Louise score > 4 including headache and/or Acute Mountain Sickness cerebral score ≥ 0.7), severe hypoxaemia (SpO_2_ < 80% for over 30 min or <75% for over 15 min), hypertensive emergency (systolic blood pressure > 200 mmHg or diastolic blood pressure > 110 mmHg), and any condition requiring any medical therapy or evacuation to LA according to the judgement of an independent physician, were also not included. Patients with one of the following conditions at baseline (LA) were not included: chronic obstructive pulmonary disease exacerbation, hypercapnia (PaCO_2_ ≥ 6 kPa), hypoxaemia at LA (SpO_2_ < 92%), uncontrolled cardiovascular disease, previous stroke, obesity (body mass index > 35 kg/m^2^), current heavy smoking (i.e. >20 cigarettes per day), renal failure, allergy to sulfonamides, and any neurologic, rheumatologic, or psychiatric diseases that could interfere with protocol compliance.

### Assessments

Detailed medical history, clinical examination, and a transthoracic Doppler echocardiography (CX 50, Philips, Philips Respironics, Zofingen, Switzerland) were performed at baseline at 760 m (LA) and directly after arrival at 3100 m (HA1) and on each consecutive day during the HA stay (HA2 and HA3) at similar times.

Echocardiographic recordings were performed using a real-time sector scanner with integrated colour, continuous wave (CW), and pulsed wave Doppler system. Recordings and measurements were performed according to the guidelines of the European Association of Cardiovascular Imaging.^17^ For each Doppler measurement, at least three measurements from all different available views were performed and the best signal was evaluated. All patients were in sinus rhythm. Maximal TRPG was calculated from maximal tricuspid regurgitation velocity (TRV_max_) obtained with CW Doppler using the modified Bernoulli equation: ΔPressure = 4× TRV_max_˄2. Right atrial pressure (RAP) was estimated taking into account the diameter of the vena cava inferior (VCId) during calm breathing and its variability during sniffing manoeuvres as following: 3 mmHg, if VCId < 2.1 cm and sniffing variability > 50%; 8 mmHg, if VCId > 2.1 cm and sniffing variability > 50%; 15 mmHg, if VCId < 2.1 cm and sniffing variability < 50%; and xx mmHg, if VCId < 2.1 cm and sniffing variability < 50%. The sPAP was calculated as TRPG + RAP. Stroke volume was estimated by the Doppler velocity time integral (VTI) method as follows: stroke volume = [left ventricular outflow tract VTI] × [cross sectional area of left ventricular outflow tract]. Cardiac output (CO) was calculated by multiplying the estimated stroke volume with the heart rate (HR). Tricuspid annular plane systolic excursion (TAPSE) was measured in M-mode. The right ventricular-arterial coupling was assessed by the TAPSE/sPAP ratio, which has been shown to be an independent predictor of invasively estimated right ventricular-arterial coupling.^18^ Areas of right atrium (RA) (end-systolic) and right ventricle (RV) (both end-systolic and end-diastolic) were manually traced in the focused four-chamber view, and the fractional area change (FAC) of RV was calculated.

The primary outcome was the change of sPAP over different time points with emphasis on the acclimatization at 3100 m. Secondary outcomes were changes of RV FAC assessing the RV transversal systolic function and changes of TAPSE assessing the RV longitudinal systolic function at different time points.

### Data analysis

All variables are presented as means ± standard deviation (SD). The differences of the means between LA and HA and HA1-HA3 are presented as mean difference and 95% confidence interval (CI).

A linear mixed model was fitted to the data with sPAP at different days. Intervention was used as fixed effects and subject as random intercept. Baseline characteristics were included in the model to control for confounding factors. We tested if weak contributing covariates could be removed from the model. Linear contrasts were defined according to the model and corrected for multiple testing by the Tukey methods. Model assumptions were tested by simulating the distribution of the data’s residuals and the random effects with Q-Q-plots and Kentucky Anscombe plots. By visual inspection of the plots, we assumed homogeneity and normality of the residuals and the random effects. A two-sided *P* < 0.05 was considered evidence for statistically significance. The statistics were performed with the statistical software R-4.3.0.

## Results

**Figure 1:**
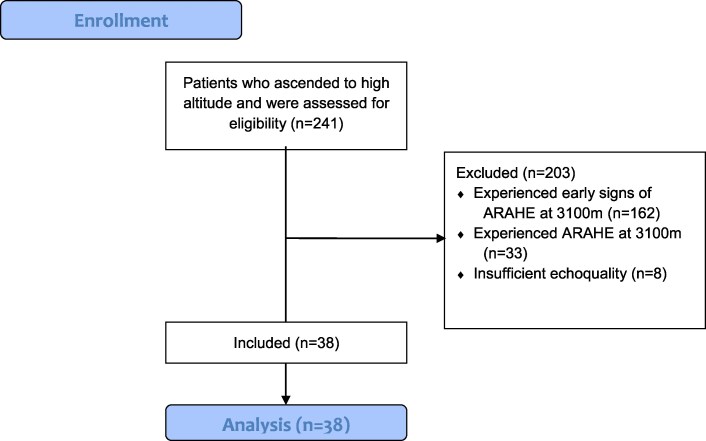
The study flow chart. Out of 241 participants who ascended to high altitude and were assessed for eligibility, 203 patients were excluded because they showed early signs of altitude related adverse health events (*n* = 162), or experienced ARAHE (*n* = 33), or had insufficient echoquality (*n* = 8). Therefore, 38 patients were included into the analysis. Adapted from CONSORT.^21^

A total of 38 patients with chronic obstructive pulmonary disease (37% female; mean age 55 ± 10 years) with mean FEV_1_ 63 ± 12%predicted were included into the analysis. The baseline characteristics are presented in *Table 1* and the study flow chart in *Figure 1*.

**Table 1:** Baseline characteristics

Number of patients	38
Gender, female/male	14/24
Age (years)	55 ± 10
Weight (kg)	734 ± 13
Height (cm)	165 ± 9
BMI (kg/m^2^)	27.0 ± 3.9
Systolic blood pressure (mmHg)	126 ± 12
Diastolic blood pressure (mmHg)	83 ± 9
Heart rate (b.p.m.)	75 ± 12
SpO_2_ (%)	95 ± 1
FEV_1_ (%predicted)	63 ± 12
FEV_1_/FVC	58 ± 9
Left ventricular ejection fraction (%)	60 ± 3
Systolic pulmonary artery pressure (mmHg)	23 ± 9
Total pulmonary resistance (WU)	5 ± 3
Cardiac output (L/min)	4.9 ± 1.3

Data are presented as means ± standard deviation.

BMI, body mass index; SpO_2_, oxygen saturation by pulse oximetry; FEV_1_, forced expiratory volume in the first second of expiration; FVC, forced vital capacity.

**Figure 2:**
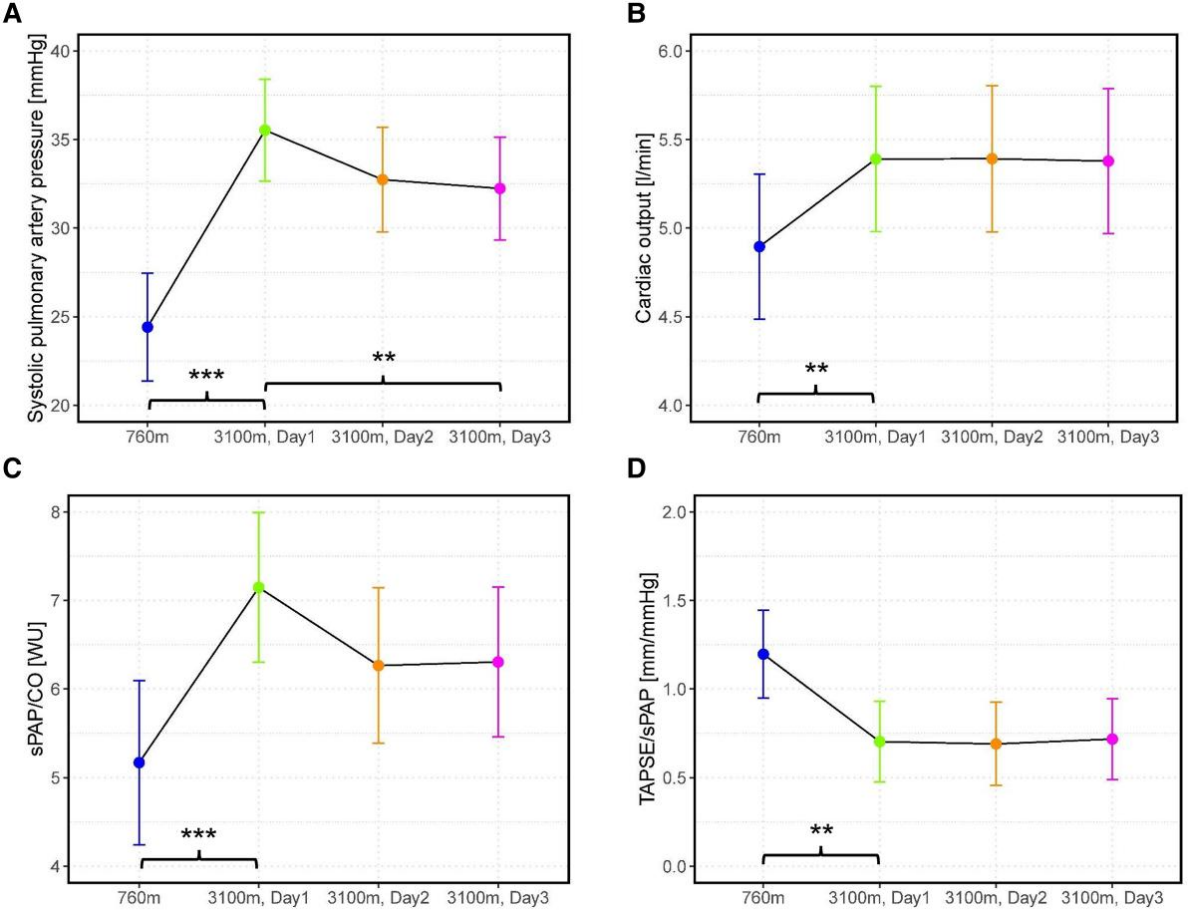
Echocardiographic measurements at 760 m and at arrival at 3100 m as well as during the 2-day high altitude stay. sPAP/CO, total pulmonary resistance; TAPSE/sPAP, right ventricular-arterial coupling. ***P < 0.001, **P < 0.01.

The sPAP increased from LA to HA1 (direct after arrival at 3100 m) by 12 mmHg (95% CI: 9–15 mmHg, *P* < 0.001) and thereafter significantly decreased during acclimatization from 35 ± 10 mmHg at HA1 to 33 ± 7 mmHg at HA2 and to 32 ± 8 mmHg at HA3 (−4 mmHg, 95% CI: −7 to −1 mmHg, *P* = 0.008) (*Figure 2* and *Table 2*). At 760 m, only 1 of the 38 patients (3%) had a TRV_max_ > 2.8 m/s, raising suspicion for clinically relevant underlying PH according to the current guidelines.^19^ After acute ascent to HA1, TRV_max_ above this cut-off was observed in 40% of our chronic obstructive pulmonary disease collective. At the last day of acclimatization, 32% of the patients still had a TRV_max_ > 2.8 m/s.

**Table 2:** Acute exposure to high altitude and acclimatization up to Day 3

	Acute altitude exposure from 760 to 3100 m				Acclimatization			
	Baseline low altitude (LA)	After arrival (HA1)	HA1-LA		Day 2 (HA2)	Day 3 (HA3)	HA3-HA1	
	Mean ± SD	Mean ± SD	Mean change (95% CI)	*P*-value	Mean ± SD	Mean ± SD	Mean change (95% CI)	*P*-value
sPAP (mmHg)	23 ± 9	35 ± 10	12 (9 to 15)	**<0.001**	33 ± 7	32 ± 8	−4 (−7 to −1)	**0**.**008**
TRV_max_ (m/s)	2.2 ± 0.5	2.8 ± 0.5	0.5 (0.3 to 0.7)	**<0**.**001**	2.7 ± 0.3	2.6 ± 0.4	−0.2 (−0.3 to −0.04)	**0**.**011**
TRPG (mmHg)	20 ± 9	32 ± 10	12 (4 to 11)	**<0**.**001**	29 ± 7	29 ± 8	−4 (−7 to −1)	**0**.**006**
RAP (mmHg)	3 ± 1	4 ± 2	1 (−0.4 to 1)	0.298	4 ± 2	4 ± 2	0 (−0.5 to 1)	0.487
sPAP/CO (WU)	5 ± 3	7 ± 3	2 (1 to 3)	**0**.**001**	6 ± 3	6 ± 3	−1 (−2 to 1)	0.156
Right ventricular-arterial coupling (TAPSE/sPAP) (mm/mmHg)	1.3 ± 1.5	0.7 ± 0.5	−0.6 (−0.9 to −0.2)	**0**.**002**	0.7 ± 0.2	0.7 ± 0.2	0 (−0.01 to 0.2)	0.116
HR (b.p.m.)	70 ± 11	77 ± 10	7 (3 to 10)	**<0**.**001**	75 ± 11	77 ± 11	0 (−3 to 3)	0.938
VTI (cm)	22.1 ± 3.4	22.7 ± 3.3	0.6 (−0.3 to 1.8)	0.172	22.4 ± 3.8	21.5 ± 3.6	−1.1 (−2.1 to −0.1)	**0**.**029**
SV (mL)	71 ± 18	69 ± 17	−2 (−5 to 5)	0.980	71 ± 15	69 ± 15	0 (−4 to 5)	0.956
CO (L/min)	4.9 ± 1.3	5.3 ± 1.3	0.4 (−0.02 to 0.9)	0.065	5.3 ± 1.2	5.3 ± 1.3	0 (−0.4 to 0.5)	0.812
EF (%)	60 ± 3	61 ± 3	1 (0.2 to 2)	**0**.**023**	60 ± 3	60 ± 3	−1 (−2 to 0.1)	0.083
TAPSE (mm)	21.0 ± 2.5	21.8 ± 2.8	0.8 (−0.1 to 1.9)	0.089	21.3 ± 2.7	21.7 ± 3.5	−0.7 (−0.1 to 0.1)	0.884
TDI’S (mm)	14.1 ± 3.1	14.4 ± 2.6	0.3 (−0.8 to 1.1)	0.435	13.7 ± 2.1	14.1 ± 2.4	−0.3 (−1.3 to 0.7)	0.514
FAC (%)	41 ± 9	41 ± 7	0 (−3 to 3)	0.891	40 ± 7	41 ± 6	0 (−3 to 3)	0.856
RA area (cm^2^)	14.4 ± 4.5	15.6 ± 4.1	1.2 (0.3 to 2.3)	**0**.**014**	15.5 ± 3.7	15.8 ± 3.8	0.2 (−0.9 to 1.2)	0.758
RV/LV	0.7 ± 0.1	0.9 ± 0.1	0.2 (−0.1 to 0.3)	0.299	0.8 ± 0.1	1.0 ± 0.1	0.1 (−0.2 to 0.4)	0.517

This table represents echocardiografic parameters at low altitude (LA, 760 m) upon arrival at 3100 m (HA1) and during consecutive days (HA2 and HA3). Data are presented as means ± standard deviation, as mean changes with corresponding 95% confidence intervals (CI) and *P*-values. Bold values indicate statistically significance.

sPAP, systolic pulmonary artery pressure; HR, heart rate; SV, stroke volume; CO, cardiac output; TDI, tissue Doppler index; FAC, fractional area change; RA, right atrium; RV, right ventricle; LV, left ventricle.

From LA to HA1, RAP and SV remained statistically unchanged, whereas the HR significantly increased by 7 b.p.m. (95% CI: 4–9 b.p.m., *P* < 0.001) and CO tend to increased, but without reaching statistical significance (0.4 L/min, 95% CI: −0.2 to 0.9, *P* = 0.065). sPAP/CO increased by 2 WU (95% CI: 1–3, *P* = 0.001) and TAPSE/sPAP decreased (−0.6 mm/mmHg, 95% CI: −0.9 to −0.2, *P* = 0.002).

Echocardiographic parameters evaluating the RV function, such as FAC and TAPSE, did not change from LA to HA. Right atrial area was significantly increased at HA (1.2 cm^2^, 95% CI: 0.3–2.3 cm^2^, *P* = 0.014).

Besides sPAP, there were no significant differences in echocardiographic parameters during acclimatization between HA1 and HA3.

### Level of acclimatization after 3 days compared to 760 m

At HA3 vs. LA, the mean sPAP was still significantly higher by 9 mmHg (5–14 mmHg, *P* < 0.001) and so were HR, RA area, and sPAP/CO, while TAPSE/sPAP was still significantly lower. All other echocardiographic parameters did not show a difference between HA3 and LA (*Table 3*).

**Table 3:** Difference to low altitude after 3 days of acclimatization

	The third day at high altitude (HA3) compared to low altitude			
	Low altitude (LA), 760 m	Third day (HA3), 3100 m	HA3-LA	
			Mean change (95% CI)	*P*-value
sPAP (mmHg)	23 ± 9	32 ± 8	9 (5 to 14)	**<0.001**
TRV_max_ (m/s)	2.3 ± 0.5	2.6 ± 0.4	0.3 (0.1 to 0.6)	**<0**.**001**
RV/RA (mmHg)	20 ± 9	29 ± 8	9 (4 to 12)	**0**.**001**
RAP (mmHg)	3 ± 1	4 ± 2	1 (−0.1 to 1)	0.067
sPAP/CO (WU)	5 ± 3	6 ± 3	1 (1 to 3)	**0**.**021**
TAPSE/sPAP (mm/mmHg)	1.3 ± 1.5	0.7 ± 0.2	−0.4 (−1.1 to −0.06)	**0**.**032**
HR (b.p.m.)	70 ± 11	77 ± 11	7 (3 to 10)	**0**.**001**
SV (mL)	71 ± 18	69 ± 15	−2 (−5 to 5)	0.915
VTI (cm)	22.1 ± 3.4	21.5 ± 3.6	−0.6 (−1.7 to 0.7)	0.399
CO (L/min)	4.9 ± 1.3	5.3 ± 1.3	0.4 (−0.01 to 0.9)	0.056
EF (%)	60 ± 3	60 ± 3	0 (−1 to 1)	0.784
TAPSE (mm)	21.0 ± 2.5	21.7 ± 3.5	0.7 (−0.7 to 2.1)	0.334
TDI’S (mm)	14.1 ± 3.1	14.1 ± 2.4	0.0 (−1.2 to 1.1)	0.878
FAC (%)	41 ± 9	41 ± 6	0 (−3 to 3)	0.774
RA area (cm^2^)	14.4 ± 4.5	15.8 ± 3.8	1.4 (0.1 to 2.8)	**0**.**045**
RV/LV	0.7 ± 0.1	1.0 ± 0.1	0.3 (−0.1 to 0.6)	0.183

This table represents echocardiographic parameters at low altitude vs. high altitude Day 3. Data are presented as means ± standard deviation, as mean changes with corresponding 95% confidence intervals and *P*-values. Bold values indicate statistically significance.

sPAP, systolic pulmonary artery pressure; HR, heart rate; SV, stroke volume; CO, cardiac output; TDI, tissue Doppler index; FAC, fractional area change; RA, right atrium; RV, right ventricle; LV, left ventricle.

## Discussion

To our knowledge, this is the first study that describes haemodynamic acclimatization up to the third day at 3100 m in lowlanders with moderate to severe chronic obstructive pulmonary disease travelling to HA who did not experience ARAHE.

We confirm previous findings of a significant increase in sPAP measured as increased TRPG with an unchanged RAP after arrival at HA.^12,14^ Although the sPAP thereafter decrease until the third day of acclimatization, it remained significantly higher than at LA. The total pulmonary resistance, the right ventricular-arterial coupling, the RA area, and the heart rate also increased after acute ascent to HA, but no short-term acclimatization process was observed.

Our observations regarding the changes in TRV_max_ and sPAP after acute exposure to HA are in line with the data from placebo arms of previous randomized clinical trials, which assessed the effects of nocturnal oxygen therapy^15^, dexamethasone^12^, and acetazolamide^13^ on pulmonary haemodynamics in patients with chronic obstructive pulmonary disease travelling to HA. Lichtblau *et al*.^14^ reported an increase of TRPG from 23 mmHg (18; 29) at LA to 32 mmHg (25; 41) at 2590 m, which corresponds to our results. In our collective, over the course of 3 days, the sPAP significantly declined as determined by a decline in TRV_max_ and calculated TRPG, with unchanged RAP. In a study^20^ evaluating cardiac acclimatization in 20 Kyrgyz healthy male lowlanders over the course of a three week exposure at 4111 m, TRPG, and subsequently sPAP, increased significantly on Day 3 at HA (change in TRPG from 21.9 mmHg at LA to 38.9 mmHg on Day 3 at HA, *P* < 0.001) and remained elevated over the entire 3-week period at HA. However, this collective was not investigated immediately after arrival. In that study, although SV decreased at HA, CO was significantly higher on Day 3 at HA compared to baseline at LA (5.9 L/min on Day 3 at HA vs. 5.1 L/min at LA, *P* < 0.01), but it returned back to LA baseline values on Day 7 at HA. In contrast, in our study, we did not observe a difference in SV or CO, although we found an expected increase in HR, which results from sympathetic activation through peripheral chemoreceptor stimulation as a response to acute hypoxia, as described in healthy individuals going to HA.^22^ The left ventricular ejection fraction was statistically significant but clinically irrelevantly increased by 1% at HA1 compared to LA.

Interestingly, we observed a statistically significant increase in the RA area after acute exposure at HA with no further significant changes during the acclimatization period. However, RA area at HA did not reach the cut-off of right atrial dilatation (RA area > 18 cm^2^) at any time point. Although Lichtblau *et al*.^13^ had similar baseline RA area measurements in patients with chronic obstructive pulmonary disease at lowland (14 ± 3 cm^2^), they did not report any change after exposure at 3100 m. Similarly, no changes in RA area were found in lowlanders with chronic obstructive pulmonary disease travelling at 2590 m.^14^ A possible mechanism for our finding could be a slight increase in tricuspid regurgitant volume, due to the elevated sPAP, leading to a statistical significant, but clinically not relevant increase in RA area. However, we did not assess the tricuspid regurgitant volume using the gold standard method of proximal isovelocity surface area; therefore, the mechanistic aspects of the observed right atrial enlargement remain unclear.

The changes in sPAP in patients with chronic obstructive pulmonary disease exposed to hypobaric hypoxia at HA are attributed to pulmonary vasoconstriction as response to alveolar hypoxia.^23^ In line, we observed a significant rise of total pulmonary resistance at HA, as estimated by sPAP/CO. In the current study, we did not assess pulmonary artery wedge pressure; therefore, a calculation of pulmonary vascular resistance (PVR) was not possible. In a former randomized, placebo-controlled trial evaluating the effects of dexamethasone on pulmonary haemodynamics in patients with chronic obstructive pulmonary disease Grade 1 or 2 (mean ± SD age 57 ± 8 years, FEV_1_ 89 ± 21%predicted) travelling to 3100 m, an increase of PVR from 1.5 ± 0.8 WU at LA to 2.4± 0.9 WU at HA was observed in the placebo group, however not statistically significant.^12^

The results of the current study indicated a reduction of right ventricular-arterial coupling, as reflected by the lower TAPSE/sPAP ratio, after exposure at 3100 m compared to baseline at lowland. The TAPSE/sPAP remained at lower levels compared to lowland during the whole acclimatization period. Despite the increase in RV afterload, the RV systolic function, expressed both by the longitudinal function through TAPSE and the FAC, did not change at HA, leading to a decrease in right ventricular-arterial coupling, with rising sPAP. This observation comes in line with the previous data from a randomized trial,^13^ in which a statistically significant reduction of TAPSE/sPAP in patients with chronic obstructive pulmonary disease acutely exposed at HA was reported (0.9 ± 0.3 at LA vs. 0.7 ± 0.3 mm/mmHg at HA, *P* < 0.05). Similar findings have been also described in lowlanders with other lung diseases; Saxer *et al*.^24^ showed that in otherwise healthy asthmatics (*n* = 22, 64% females, mean age 44 ± 12 years) exposed to 3100 m, TAPSE/sPAP decreased significantly by −0.2 mm/mmHg (−0.3 to −0.1, *P* < 0.001) at Day 2 at HA compared to LA and the right ventricular-arterial coupling remained significantly reduced by −0.2 mm/mmHg (−0.3 to 0.1, *P* = 0.002) compared to lowland at Day 21 at HA. The significance of lack of increase in RV systolic function for the maintenance of right ventricular-arterial coupling under conditions of increased RV afterload and its role in acclimatization of patients with chronic obstructive pulmonary disease at HA requires further investigation.

Despite the alternations in pulmonary haemodynamics described above, in our chronic obstructive pulmonary disease cohort, the acute ascent and the 3-day stay at 3100 m were tolerated well by definition as only patients without developing any ARAHE were included; nevertheless, 40% of the patients developed a relevant rise in sPAP after arriving at HA. In the 2-day acclimatization period, sPAP quickly decreased, but it never reached the LA values. Short-term acclimatization did not affect the total pulmonary resistance or any other of the echocardiographic parameters. As the patients included in our study were asymptomatic at HA, it remains interesting to further investigate the underlying pathophysiological mechanisms leading to ARAHE in addition to changes in pulmonary haemodynamics, which have been well tolerated by our chronic obstructive pulmonary disease cohort.

### Limitations

Although right heart catheterization is the gold standard to access pulmonary haemodynamics and to diagnose PH, this is not repeatedly feasible and especially not at our HA facilities. However, transthoracic echocardiography has been shown to correlate closely with the invasive measurements at HA.^25^ Performing an echocardiography in patients with chronic obstructive pulmonary disease can often be challenging due to thoracic hyperinflation. In our study, we achieved sufficient echocardiography quality in the vast majority of patients, most probably related to the fact that we did not include patients with severe chronic obstructive pulmonary disease likely to have relevant over inflation due to emphysema and echocardiography was done and interpreted by adequately trained investigators. The current trial is a prospective study in a predefined collective; thus, the results must be implicated with caution. Patients with very severe chronic obstructive pulmonary disease or hypoxia/hypercapnia at rest were excluded from our study for safety reasons. Acclimatization was accessed only during a time period of 2 days; a longer acclimatization time may have other effects on pulmonary haemodynamics. Due to logistic issues, we were not able to perform a last echocardiographic assessment after descent to lowland; therefore, no conclusions on reversibility of the observed changes can be made. Moreover, there is a selection bias present, as we only included patients who did not develop any ARAHE, as described in the study design section above. Therefore, generalizability of these findings might be difficult and may only apply to patients with chronic obstructive pulmonary disease without ARAHE, making extrapolation to patients with chronic obstructive pulmonary disease developing AMS not possible. Further randomized trials accessing the effects of acute exposure and short-term acclimatization at HA in patients with chronic obstructive pulmonary disease are needed.

## Conclusion

In stable patients with moderate to severe chronic obstructive pulmonary disease, travelling to 3100 m and not revealing acute mountain sickness, relevant hypoxaemia, or other adverse events, the sPAP initially increased, along with an elevated total pulmonary resistance and impaired RV ventriculo-arterial coupling. Although sPAP quickly decreased during 2 days of acclimatization, it still remained significantly higher compared to baseline measurements at LA. We did not find evidence that short-term acclimatization influenced any other echocardiographic parameters.

## Data Availability

The raw data, which have been analysed and supported the findings of this article, can be made available from the corresponding author after request.

## Lead author biography

Konstantinos Bitos is an internal medicine specialist currently serving as a cardiology resident at the University Heart Center Zurich, with a particular focus on heart failure and cardiovascular imaging. He is deeply involved in clinical research in the field of cardiology and cardiovascular medicine as well as in HA research.

Julian Müller is a medical scientist currently holding a post-doc position at the Department of Pulmonology in the University Hospital Zurich, Switzerland. His research focuses on pulmonary vascular disease, exercise physiology, exercise haemodynamics, and HA research.

Adilet Omuralieva is a cardiologist and researcher with special interest on echocardiography and HA medicine at the Department of Cardiology at the National Center of Cardiology and Internal Medicine Named after Academician M. Mirrakhimov in Bishkek, Kyrgyzstan.
